# 3D Yolk–Shell Structured Si/void/rGO Free-Standing Electrode for Lithium-Ion Battery

**DOI:** 10.3390/ma14112836

**Published:** 2021-05-26

**Authors:** Jin Shao, Yi Yang, Xiaoyan Zhang, Liming Shen, Ningzhong Bao

**Affiliations:** 1 State Key Laboratory of Material-Oriented Chemical Engineering, College of Chemical Engineering, Nanjing Tech University, Nanjing 210009, China; 201861104082@njtech.edu.cn (J.S.); 201861104037@njtech.edu.cn (Y.Y.); lshen@njtech.edu.cn (L.S.); 2 Zigong Innovation Center of Zhejiang University, Zhejiang University, Zigong 643000, China

**Keywords:** lithium-ion battery, silicon anode, graphene, flexible, free-standing

## Abstract

In this study, we have successfully prepared a free-standing Si/void/rGO yolk–shell structured electrode via the electrostatic self-assembly using protonated chitosan. When graphene oxide (GO) is dispersed in water, its carboxyl and hydroxyl groups on the surface are ionized, resulting in the high electronegativity of GO. Meanwhile, chitosan monomer contains -NH_2_ and -OH groups, forming highly electropositive protonated chitosan in acidic medium. During the electrostatic interaction between GO and chitosan, which results in a rapid coagulation phenomenon, Si/SiO_2_ nanoparticles dispersed in GO can be uniformly encapsulated between GO sheets. The free-standing Si/void/rGO film can be obtained by freeze-drying, high-pressure compression, thermal reduction and HF etching technology. Our investigation shows that after 200 charge/discharge cycles at the current density of 200 mA·g^−1^, the specific discharge capacity of the free-standing electrode remains at 1129.2 mAh·g^−1^. When the current density is increased to 4000 mA·g^−1^, the electrode still has a specific capacity of 469.2 mAh·g^−1^, showing good rate performance. This free-standing electrode with a yolk–shell structure shows potential applications in the field of flexible lithium-ion batteries.

## 1. Introduction

Lithium-ion batteries (LIBs) have received extensive attention due to their large capacity, high energy density, high charge and discharge efficiency, long cycle life, and environmental friendliness [1,2,3,4,5,6]. At present, the anode material of LIBs is mainly graphite, which has good conductivity and stability. However, the relatively low specific capacity (372 mAh·g^−1^) and rate performance of commercial graphite anode materials are increasingly difficult to meet the requirements of large-scale power supplies [7,8]. Therefore, silicon-based, tin-based and titanium-based anode materials for LIBs have become a research hotspot. Among them, silicon (Si) is considered to be the most ideal new generation anode material because of its abundant content in the Earth’s crust, low delithiation potential (0.5 V) and high theoretical specific capacity (4200 mAh·g^−1^) [9,10,11]. However, Si anodes have inherent problems such as low conductivity, large volume changes (about 300%) during charge and discharge, and the formation of unstable solid electrolyte interface (SEI) films [12]. This leads to poor rate performance, fast capacity decay and low coulomb efficiency of Si-based anodes. In order to reduce the volume expansion effect of the Si anode, nano-Si materials with different morphologies, such as porous Si nanoparticles [13,14], Si nanowires [15], and Si thin films [16,17], etc., have been studied. The size effect relieves volume expansion to a certain extent and improves the cycle stability of the electrode. In addition, studies have shown that encapsulating Si in a carbon matrix, such as graphene and porous carbon, can effectively alleviate the volume expansion of Si and increase the electrical conductivity [18,19].

Besides, during the typical preparation process of electrodes for LIBs, the active substances usually need to be blended with polymer binders and conductive additives, followed by a long time vacuum drying. The addition of polymer binders will inevitably hinder the ion transport, and thus reduce the electrode conductivity. Meanwhile, the active material can fall off the current collector due to its large volume expansion during the charge/discharge cycle, resulting in a decrease in capacity [20,21,22]. In contrast to the complex preparation process of electrodes, a free-standing electrode can be directly prepared to be used as the working electrode [23]. With the characteristics of good mechanical properties, environmental friendliness, and ease of preparation, free-standing electrodes are considered as the key materials for the preparation of flexible lithium-ion batteries. At present, Zhang et al. [24] have prepared a flexible self-supporting silicon-based composite film by suction filtration. The discharge specific capacity after 80 cycles at a current density of 200 mA·g^−1^ is maintained at 1209.3 mAh·g^−1^. How to further inhibit the volume expansion of Si and improve the cycle stability of the battery is still the focus and difficulty of this field.

In this study, we have successfully prepared a free-standing Si/void/rGO yolk–shell structured electrode via electrostatic self-assembly using protonated chitosan. With the electrostatic interaction between graphene oxide (GO) and chitosan, which results in a rapid coagulation phenomenon, Si/SiO_2_ nanoparticles dispersed in GO can be uniformly encapsulated between GO sheets [25]. The free-standing Si/void/rGO film can be obtained by freeze-drying, high-pressure compression, thermal reduction and HF etching technology. Our investigation shows that the unique yolk–shell structure of the composite electrode, and the excellent conductivity and flexibility of graphene, can effectively prevent the destruction of the electrode structure caused by the huge volume expansion of Si during the charge/discharge process. In addition, the integrity of the electrode maintained by the free-standing structure not only provides a continuous conductive path for the Si nanoparticles, but also effectively stops the active material from falling off the current collector. After 200 charge/discharge cycles at the current density of 200 mA·g^−1^, the specific discharge capacity of the free-standing electrode remains at 1129.2 mAh·g^−1^. When the current density is increased to 4000 mA·g^−1^, the electrode still has a specific capacity of 469.2 mAh·g^−1^, showing a good rate performance. This free-standing electrode with a yolk–shell structure shows potential applications in the field of flexible lithium-ion batteries.

## 2. Materials and Methods

### 2.1. Preparation of Si/SiO_2_/GO Dispersion

The modified hummers method was used to prepare GO according to the literature reports [26,27], and a 3 g/L GO dispersion was prepared with deionized water. The nano-silicon powders (20–60 nm, 99.9%, Aladdin) were heated in a tube furnace at a rate of 5 °C/min to 700 °C and kept for 2 h to obtain the Si/SiO_2_ composites. Then 60 mg Si/SiO_2_ particles were added to 20 mL of GO dispersion and ultrasonicated for 5 min to obtain the Si/SiO_2_/GO dispersion.

### 2.2. Preparation of Si/SiO_2_/rGO Film

Acetic acid (2 mL) (99.5%, Miacklin) was added into 50 mL of deionized water. After stirring evenly, 1 g chitosan (95%, Miacklin) was added into the water and stirred until the chitosan was completely dissolved to obtain a coagulation bath. The above Si/SiO_2_/GO dispersion was slowly dropped into the coagulation bath under vigorous stirring, and continued stirring for 20 min. The obtained precipitate was centrifuged at 2000 rpm, washed three times with deionized water, and transferred to a watch glass. Then, it was freeze-dried for 12 h to obtain Si/SiO_2_/GO aerogel. A pressure of 6 MPa was applied to obtain a smooth and dense film. The film was annealed in argon at 900 °C for 2 h to obtain a Si/SiO_2_/rGO film.

### 2.3. Preparation of Free-Standing Si/void/rGO Electrode

In order to remove the SiO_2_ layer on the Si surface, the Si/SiO_2_/rGO film was immersed in HF solution (HF:ethanol:H_2_O = 3.5:2:1.5, volume ratio) and kept at room temperature for 30 min. After three times of ethanol immersion to remove the residual HF solution, the etched film was dried in a vacuum drying oven at 60 °C for 6 h to obtain the free-standing Si/void/rGO film. After cutting, free-standing Si/void/rGO electrode was obtained. For comparison, Si particles were directly added to the GO dispersion, the other steps were the same as before except for HF etching, and the Si/rGO electrode was obtained.

### 2.4. Materials Characterization

The crystalline phases of samples were characterized by X-ray diffraction (XRD) using a Rigaku SmartLab diffractometer (Cu Kα radiation, λ = 1.5418, 40 kV, 100 mA, from 2*θ* = 10°–90°, scan-speed 0.8 s/step, and increment of 0.02 deg/s, Tokyo, Japan). The morphology and microstructure were characterized by field emission scanning electron microscopy (FESEM, Nova NanoSEM 450, Hillsboro, OR, USA). Raman spectra were collected with Raman laser spectrometer (Renishaw inVia, London, England) using 514.5 nm laser excitation. Thermogravimetric analysis (TGA) was performed on a Netzsch STA449F5 thermogravimetric analyzer (Selb, Germany). The samples (2.129 mg) were heated in oxygen from room temperature to 800 °C at 10 °C/min. X-ray photoelectron spectroscopy (XPS) analysis was performed on a PHI-5000 VersaProbe X-ray photoelectron spectrometer (Kanagawa, Japan). The specific surface areas were analyzed with the Brunauer–Emmett–Teller (BET) method using a BELSORP-mini nitrogen adsorption apparatus (Osaka, Japan), and the pore size distribution by the Barrett–Joyner–Halenda (BJH) method.

### 2.5. Electrochemical Analysis

The electrochemical test cells were assembled in an argon-filled glove box (vigor, SG1200/750TS) in which H_2_O and O_2_ concentration were kept below 0.1 ppm. Si/void/rGO film electrode and Si/rGO electrode samples were cut into circular electrodes with a diameter of 12 mm and directly used as working electrodes without adding any conductive agent or binder. The mass of each piece of Si/void/rGO film electrode and Si/rGO electrode was about 2 mg and 2.5 mg. In the Si/void/rGO film electrode, Si accounts for 48.45% and the mass was 0.969 mg. The bare Si anode was fabricated by mixing the Si powders, acetylene black (as a conductive agent), and polyvinylidene fluoride (as a binder) in N-methyl-2-pyrrolidone at a weight ratio of 8:1:1. The slurry was uniformly plastered on a copper foil current collector via doctor blade processing, followed by vacuum drying at 80 °C for 12 h. The specific capacity has been calculated based on the mass of Si. The CR2032 coin cells with a lithium metal counter electrode and a polypropylene separator (Celgard2400) were used to assess the electrochemical performance. The electrolyte was 1.0 M LiPF_6_ and fluoroethylene carbonate (10%, FEC) was dissolved in a mixture of ethylene carbonate (EC), dimethyl carbonate (DMC) and ethyl methyl carbonate (EMC) (1:1:1, volume ratio). The galvanostatic charge/discharge measurements were carried out on Neware, BTS-3000 in the voltage range of 2.0 to 0.01 V (vs. Li/Li^+^) at room temperature (25 ± 1 °C). The cyclic voltammograms (CV) and electrochemical impedance spectroscopy (EIS) were measured on an electrochemical workstation (CHI660D) at a scanning rate of 0.1 mV/s. The EIS were recorded by applying an AC voltage of 10 mV amplitude over the frequency range from 100 kHz to 0.01 Hz

## 3. Results and Discussion

**Figure 1:**
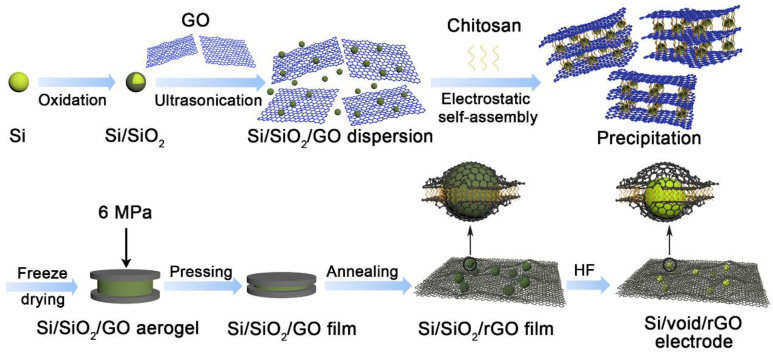
Schematic illustration of the fabrication process of free-standing Si/void/rGO electrode.

The schematic illustration of the preparation process of the free-standing Si/void/rGO electrode is shown in Figure 1. Firstly, a layer of SiO_2_ was prepared on the outer surface of the Si nanoparticles through high-temperature oxidation to obtain a Si/SiO_2_ composite structure. The SiO_2_ of the outer layer not only has good hydrophilicity, which can improve the dispersibility of Si/SiO_2_ in GO, but also can be used as a template for voids. Then, the Si/SiO_2_ was uniformly dispersed in GO by ultrasound and slowly dropped into the chitosan coagulation bath. It was observed that the Si/SiO_2_/GO dispersion was precipitated in the chitosan coagulation bath. This is mainly due to the reason that when GO is dispersed in water, the -OH and -COOH groups on the surface are ionized, resulting in high electronegativity. The chitosan monomer contains -NH_2_ and -OH groups, which form highly electropositive protonated chitosan in acidic medium. It reacts electrostatically with GO, pulling graphene sheets close to each other, resulting in a rapid coagulation phenomenon. At the same time, the Si/SiO_2_ nanoparticles were encapsulated between the layers of GO to obtain a flocculent precipitate of the Si/SiO_2_/GO composite structure. The flocculent precipitate was made into a Si/SiO_2_/GO aerogel by freeze-drying, and then through simple physical pressure, and annealing in argon at 900 °C for 2 h, a Si/SiO_2_/rGO electrode film can be obtained. Finally, HF solution was used to etch away the SiO_2_ template on the Si surface to obtain a free-standing Si/void/rGO electrode. Through the etching, voids can be formed around the Si nanoparticles to buffer the stress caused by the volume expansion of Si during the charge and discharge process. The reduced graphene oxides have excellent conductivity and are cross-linked with each other to form a conductive network, which greatly improves the overall conductivity of the composite material. At the same time, graphene has good mechanical properties and flexibility, which can play a role in mechanical support to prepare free-standing electrodes.

### 3.1. Structural Characterization

**Figure 2:**
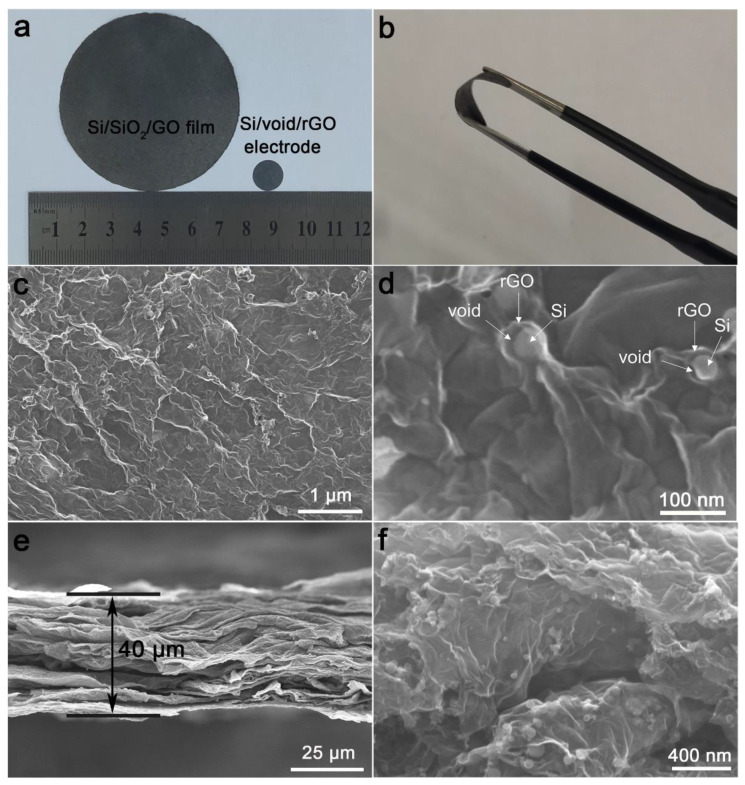
(**a**,**b**) Photographs of Si/SiO_2_/GO aerogel after compression and free-standing Si/void/rGO electrode; (**c**,**d**) surface SEM images of the free-standing Si/void/rGO electrode at different magnifications; (**e**,**f**) cross-section SEM images of the free-standing Si/void/rGO electrode at different magnifications.

The photographs of the Si/SiO_2_/GO aerogel after compression and free-standing Si/void/rGO electrode are shown in Figure 2a. After the aerogel is subjected to a simple physical pressure treatment, the surface becomes smoother and forms a film-like structure with a diameter of 65 mm. The Si/SiO_2_/GO film was cut into a 12-mm diameter wafer, which was directly used as the working electrode after further annealing, etching and drying. It can be observed that the surface of the electrode is relatively smooth without any obvious defects. The SEM images of the surface and cross-section view of the free-standing Si/void/rGO electrode are shown in Figure 2c–f. The surface (Figure 2c) and cross-section (Figure 2e) SEM images at low magnification clearly show that the material as a whole is formed by a large number of graphene sheets interconnected and closely stacked. The thickness of the electrode sheet is about 40 μm, and the Si nanoparticles are coated in the graphene sheets. The SEM image at a high magnification (Figure 2d) shows the microtopography of a single Si/void/rGO composite structure on the surface of the free-standing electrode. The nano-Si particles are wrapped by graphene, and there is a void between the nano-Si and the graphene layer, thereby forming a yolk–shell structure. From the SEM image (Figure 2f) of the cross-section at a high magnification, it can be seen that the nano-Si particles are uniformly dispersed in the graphene without obvious agglomeration, indicating that the Si nanoparticles in the material have good dispersibility. Therefore, we have successfully prepared a flexible self-supporting Si/void/rGO electrode with a yolk–shell structure. This structure reserves a certain space for the volume expansion of Si, which plays as a buffer layer and reduces the damage to the electrode material caused by the volume expansion of Si during the charge and discharge process. At the same time, the cross-linked graphene provides a conductive network, which improves the overall conductivity of the material.

**Figure 3:**
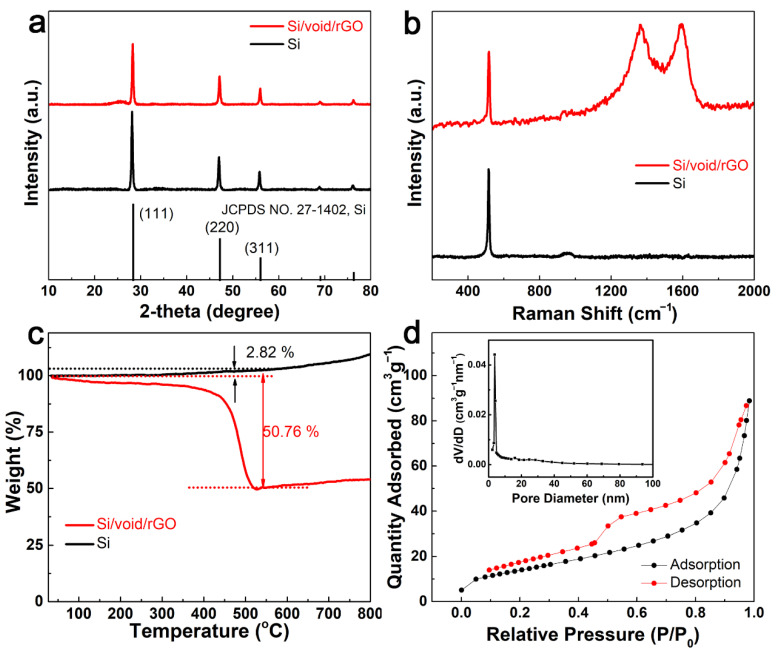
(**a**) XRD patterns and (**b**) Raman scattering spectra of free-standing Si/void/rGO electrode and Si; (**c**) TG curve and (**d**) N_2_ adsorption–desorption isotherm and the corresponding pore size distribution of free-standing Si/void/rGO electrode.

Figure 3a is the X-ray diffraction (XRD) patterns of Si powder sample and the free-standing Si/void/rGO electrode. It can be seen that the XRD patterns of the Si powder and the free-standing Si/void/rGO electrode are similar, and the diffraction peaks at 28, 47, and 56° correspond to the (111), (220) and (311) crystal planes of Si, respectively, which match well with the standard XRD pattern [28]. In addition, the XRD peak of the free-standing Si/void/rGO electrode is sharp, indicating that the silicon in the composite material has good crystallinity, and the thermal reduction process did not destroy the structure of the nano-silicon. In the XRD pattern of the free-standing Si/void/rGO electrode, the small packet peak at 2θ = 25° is the characteristic diffraction peak of graphene, indicating the existence of graphene.

Figure 3b shows the Raman spectra of the free-standing Si/void/rGO electrode and Si samples. From the Raman spectra of the Si/void/rGO film electrode, we can clearly see the characteristic D peak (1350 cm^−1^), G peak (1595 cm^−1^), and the characteristic peak of Si (516 cm^−1^). The D peak can be attributed to structural defects, while the G peak corresponds to the sp^2^ carbon structure [29,30]. The Si characteristic peak of the Si/void/rGO film electrode sample is the same as the characteristic peak of pure Si, indicating that the preparation process did not destroy the Si structure.

Figure 3c shows the thermogravimetric analysis (TGA) curve of the free-standing Si/void/rGO electrode composite sample and the pure Si sample under oxygen atmosphere. The composite material sample is mainly composed of Si and carbon (amorphous carbon formed by the carbonization of chitosan and reduced graphene oxide), and its content can be quantified by TGA analysis. From room temperature to 400 °C, the mass of the sample drops slowly, which may be related to the removal of a small amount of water in the sample and the pre-combustion of carbon. The mass reduction between 400 °C and 550 °C is due to the oxidation of the carbon structure. [31,32]. The TG curve of the pure Si sample began to rise slowly at 350 °C, at which time a small amount of Si was oxidized to form SiO_2_. At about 550 °C, the weight no longer decreased, and there was an upward trend. This means that the carbon in the sample had been converted to carbon dioxide, and more Si continued to be oxidized to form SiO_2_. Therefore, the mass of Si and C can be calculated at 550 °C. At this temperature, the weight percentage of the pure Si samples increased by 2.82%, and the weight percentage of the composite samples decreased by 50.76%. Assuming that the weight percentages of Si and C are X and Y, respectively, by calculating the following equations: X + Y = 1, 1.0282X/(1.0282X + Y) = 1 − 0.5076, it can be concluded that the contents of Si and C were 48.45% and 51.55%, respectively. [33]

Figure 3d shows the N_2_ adsorption–desorption isotherm and the corresponding pore size distribution of the free-standing Si/void/rGO electrode. The N_2_ adsorption–desorption isotherm curve conforms to the characteristics of the IV curve. According to the Barrett–Joyner–Halenda (BJH) equation, the specific surface area of the free-standing Si/void/rGO electrode is 75.7 m^2^ g^−1^, the pore size range is 2–100 nm, and most pore sizes are around 3.7 nm. These mesoporous structures enable a larger contact area between the electrolyte and the electrode, and the diffusion channel for the insertion of lithium ions is shorter, and the overall electrochemical performance of the material is improved [34].

**Figure 4:**
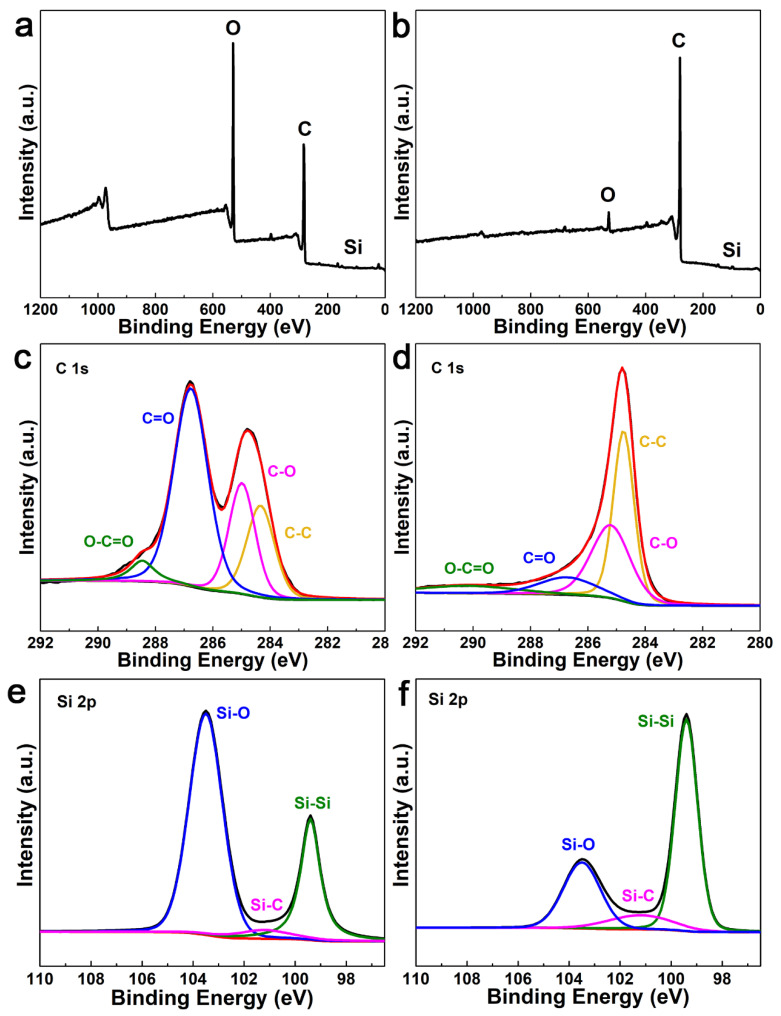
XPS spectra of (**a**) Si/SiO_2_/GO film and (**b**) free-standing Si/void/rGO electrode; C1s curve of (**c**) Si/SiO_2_/GO film and (**d**) free-standing Si/void/rGO electrode; Si 2p curve of (**e**) Si/SiO_2_/GO film and (**f**) free-standing Si/void/rGO electrode.

The X-ray photoelectron spectroscopy (XPS) was used to further characterize the chemical state of carbon in the Si/void/rGO composite electrode. Figure 4a,b are the XPS spectra of the Si/SiO_2_/GO film and free-standing Si/void/rGO electrode samples. The spectra show that the Si/SiO_2_/GO film and the free-standing Si/void/rGO electrode both contain the three elements of Si, O and C. Figure 4c,d are the C1s spectra of the Si/SiO_2_/GO film and the free-standing Si/void/rGO electrode, showing the presence of different oxygen-containing functional groups in the sample. The four peaks at 284.0, 285.2, 288 and 289.2 eV correspond to the C=C/C–C, C–O, C=O and COOH groups, respectively [35,36]. However, compared with the Si/SiO_2_/GO film, the peak intensity of the oxygen-containing functional groups of the free-standing Si/void/rGO electrode composite is significantly weakened, which indicates that thermal reduction at 900 °C for 2 h can effectively remove most of the oxygen-containing functional groups of GO, which improves the conductivity of the composite electrode. As shown in Figure 4e, the Si 2p spectrum of the Si/SiO_2_/GO film can be deconvolved into three contributions. One dominant peak at 103.5 eV corresponds to the bonding of Si–O. The second peak located at 103.5 eV corresponds to the bonding of Si–Si. The third relatively weak peak located at 101.2 eV is attributed to a very small amount of Si–C [37]. The Si 2p spectrum of the free-standing Si/void/rGO electrode is shown in Figure 4f. The positions of the three peaks are still the same as before, but the strength of the peaks change accordingly. The peak of the Si–Si bond is significantly enhanced with an obvious decreased peak intensity of the Si–O bond, indicating that most of the SiO_2_ had been removed after the HF treatment. In addition, the peak of the Si–C bond had increased, indicating that the interaction between Si and C was enhanced by calcining, which was beneficial to improve the conductivity of the material.

### 3.2. Evaluation of Electrochemical Performance

**Figure 5:**
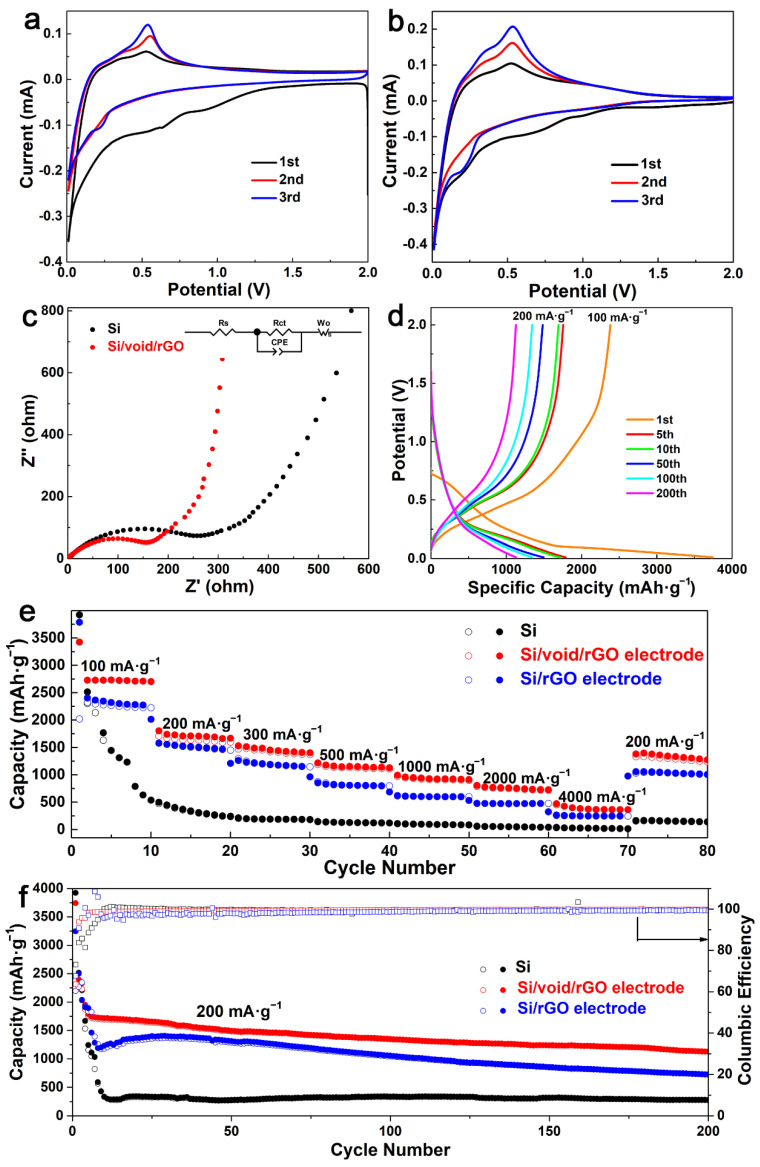
Cyclic voltammetry (CV) curves of the (**a**) free-standing Si/void/rGO electrode and (**b**) bare Si anode, (**c**) electrochemical impedance spectroscopy (EIS) spectrum and fitted equivalent circuit diagram, (**d**) charge/discharge profiles of the free-standing Si/void/rGO electrode at 1st, 5th, 10th, 50th, 100th and 200th cycles, (**e**) rate performance at various current densities, and (**f**) cycling performance and coulombic efficiency (CE) at 200 mA·g^−1^ of the free-standing Si/void/rGO electrode, Si/rGO electrode and bare Si electrode.

In order to analyze the oxidation–reduction reaction of the electrode, the cyclic voltammetry (CV) curves of the free-standing Si/void/rGO electrode and bare Si anode were measured in the voltage range of 0.01 V to 2 V at a scanning rate of 0.01 mV/s, as shown in Figure 5a,b. In the first scan, the free-standing Si/void/rGO electrode shows two broad reduction peaks at 0.7 V and 1.13 V, which disappear in the subsequent cycles. This is mainly due to the formation of a solid electrolyte interface (SEI) film at the electrode/electrolyte interface [38]. There is a reduction peak at 0.2 V, which is due to the a–Li*_x_*Si alloy phase formed by the insertion of Li^+^ into Si [39]. Due to the delithiation reaction of the a–Li*_x_*Si alloy phase and the formation of amorphous silicon (a–Si), the free-standing Si/void/rGO electrode shows oxidation peaks at 0.31 V and 0.51 V [40]. By comparing these three CV curves, the intensities of the reduction peak and oxidation peak both gradually increase with the number of cycles. This is mainly due to the gradual activation of Si during the intercalation/deintercalation of lithium ions. The electrolyte wetting issue is one of the reasons of this situation, and the other reason is that there is an activation process on account of the solid-state lithium ions diffusing in the internal silicon [32]. The CV curves of the second circle and the third circle almost coincide, indicating that the charge/discharge process is highly reversible.

The electrochemical performance of the free-standing Si/void/rGO electrode sample was further characterized by electrochemical impedance spectroscopy (EIS), as shown in Figure 5c. The Nyquist diagram includes a semicircle in the high-frequency region and a diagonal line in the low-frequency region. The flat semicircle in the high-frequency region reflects the charge transfer resistance (R_ct_) on the electrode surface, while the corresponding slope of the diagonal line in the low-frequency region reflects the Warburg impedance (Z_w_) of the lithium-ion diffusion rate inside the electrode [41]. Figure 5c shows that the free-standing Si/void/rGO electrode has a smaller high-frequency semicircle diameter and a higher low-frequency line slope (R_ct_ = 182.7 Ω, R_s_ = 1.03 Ω) than the pure Si materials (R_ct_ = 307.6 Ω, R_s_ = 2.13 Ω). These results are verified by the fitted data based on the equivalent circuit [42], so the free-standing Si/void/rGO electrode has lower charge transfer resistance and ion diffusion resistance.

The charge/discharge profiles of the free-standing Si/void/rGO electrode are shown in Figure 5d. The current density of the first cycle charge/discharge curves is 100 mA·g^−1^, and the current density of the other charge/discharge curves are 200 mA·g^−1^. The first charge/discharge specific capacity is 2381.6 and 3742.2 mAh·g^−1^, respectively. The main reason for the capacity loss is the formation of an SEI film on the surface of the electrode, which consumes a large number of lithium ions. There are also some Si particles that are unavoidably cracked or crushed due to the stress generated by the expansion during the first charge and discharge process, resulting in a large amount of irreversible capacity. It could also be attributed to electrode polarization and electrolyte decomposition [43]. In the subsequent charge and discharge cycles, the free-standing Si/void/rGO electrode showed excellent cycle performance. After 100 cycles, the specific charge/discharge capacity of the electrode was 1343.5 and 1439.3 mAh·g^−1^, respectively. After 200 cycles of cycles, the specific charge/discharge capacity of the electrode still remained at 1127.3 and 1129.2 mAh·g^−1^, respectively.

The rate performance of the free-standing Si/void/rGO electrode, the Si/rGO electrode and the pure Si electrode at various current densities is shown in Figure 5e. When the pure Si electrode was cycled 10 times at a current density of 100 mA·g^−1^, the first cycle charge/discharge specific capacity of the battery was relatively high, which was close to the theoretical specific capacity. However, the performance of the battery was unstable, and the specific capacity decreased rapidly as the number of charge/discharge increased. Subsequently, the current densities of 200, 300, 500, 1000, 2000 and 4000 mA·g^−1^ were used for testing, and the specific discharge capacities were 242.6, 183.5, 119.6, 84.9, 40.9, and 14.8 mAh·g^−1^. When the current density returned to 200 mA·g^−1^, the specific capacity was only 141.5 mAh·g^−1^. The Si/rGO electrode was tested at the above current density, and its specific capacities were 2222.6, 1450.3, 1146.1, 796.5, 599.4, 475.7, and 247.2 mAh·g^−1^. When the current density returned to 200 mA·g^−1^, the specific capacity was only 1004.1 mAh·g^−1^. The free-standing Si/void/rGO electrode was cycled 10 times at a current density of 100 mA·g^−1^, and the discharge specific capacity was maintained at about 2750 mAh·g^−1^. Then the current density was increased, and the specific capacities were 1803.6, 1528.8, 1217.8, 993.4, 804.1, and 469.2 mAh·g^−1^, respectively. When the current density returned to 200 mA·g^−1^, the specific capacity could reach 1382.2 mAh·g^−1^. The above results indicate that the free-standing Si/void/rGO electrode has excellent rate performance and the formation of void between Si and rGO can effectively reduce the damage to the structure caused by volume changes.

Figure 5f shows the cycling performance of the free-standing Si/void/rGO electrode, Si/rGO electrode and pure Si electrode sample after being activated at a current density of 100 mA·g^−1^, and then cycling for 200 times at a current density of 200 mA·g^−1^. Although the specific discharge capacity of the pure Si sample reached 3922.1 mAh·g^−1^ in the first cycle, the specific capacity decayed to 337.1 mAh·g^−1^ after 10 cycles. The main reason is that the conductivity of the pure Si electrode is low, and there exists huge volume changes during charging and discharging. The stress generated by this process causes the cracking and crushing of Si particles, which in turn leads to the formation of an unstable SEI film and consumes a large amount of Li^+^. It can even cause the active material to fall off from the current collector, which further increases the generation of an unavailable capacity, resulting in a significant decrease in the capacity. After the Si/rGO electrode was cycled for 200 cycles at a current density of 200 mA·g^−1^, the specific capacity remained only 727.2 mAh·g^−1^. The initial discharge capacity of the free-standing Si/void/rGO electrode sample was 3742.2 mAh·g^−1^. In the following cycles, the capacity decayed more obviously, mainly due to the formation of the unstable SEI film in the early stage. Compared with the performance of the first five cycles, the capacity reduction rate from the 5th to the 200th cycle gradually slowed down, maintaining a relatively slow downward trend. After 200 cycles, the discharge-specific capacity remained at 1129.2 mAh·g^−1^, and the coulombic efficiency was 99.8%.

Recently, a three-dimensional coral-like Si nanostructure coated with C/rGO (CL-Si@C/rGO) was prepared by Wang et al. [44] through the high-temperature magnesiothermic reduction of SiO_2_ nanotubes. The CL-Si@C/rGO composite can deliver a reversible capacity of 739.1 mAh·g^−1^ at a high current density of 2 A· g^−1^. Zhang et al. [24] have prepared a free-standing electrode by suction filtration. The discharge specific capacity after 80 cycles at a current density of 200 mA·g^−1^ is maintained at 1209.3 mAh·g^−1^. Recently, Zeng et al. [45] designed and synthesized the free-standing N-doped porous carbon nanofibers sheathed pumpkin-like Si/C composites (Si/C-ZIF-8/CNFs), which deliver a reversible capacity of 945.5 mAh·g^−1^ at 200 mA g^−1^, with a capacity retention of 64% for 150 cycles. Compared with these results, the cycling performance of the free-standing Si/void/rGO electrode we studied is more stable. It also has excellent rate performance, and its specific capacity is 804.1 mAh·g^−1^ at a current density of 2 A·g^−1^. The free-standing Si/void/rGO electrode had appropriate buffer gaps and was embedded between the graphene layers, which can make Si expand and contract inside the mesoporous carbon shell. Moreover, as a flexible free-standing structure, it avoids the use of a binder and conductive agent. Therefore, it can maintain the integrity of the electrode structure and had an excellent cycling performance.

## 4. Conclusions

In this study, we have successfully prepared a free-standing Si/void/rGO yolk–shell structured electrode via electrostatic self-assembly using protonated chitosan. The free-standing composite electrode has high flexibility and good mechanical properties, maintains the integrity of the electrode, and can be directly used as a working electrode of a lithium-ion battery without adding other binders and conductive agents. The Si nanoparticles are evenly coated between the graphene sheets, which effectively avoids direct contact between the Si and the electrolyte, and promotes the formation of a stable SEI film. At the same time, graphene has good flexibility and conductivity, which can buffer the volume expansion of Si and improve the conductivity of composite materials. In addition, in the yolk–shell structure, the space reserved around the Si particles can further buffer the damage of the structure caused by the huge volume change in Si during the charge/discharge process, thereby improving the cycle performance of the battery.

## Data Availability

The data presented in this study are available on request from the corresponding author.
